# The State-Trait Model of Cheerfulness: Tests of Measurement Invariance and Latent Mean Differences in European and Chinese Canadian Students

**DOI:** 10.5964/ejop.3003

**Published:** 2022-05-31

**Authors:** Chloe Lau, Francesca Chiesi, Donald H. Saklofske

**Affiliations:** 1 Department of Psychology, University of Western Ontario, London, Canada; 2 Department of Neuroscience, Psychology, Drug, and Child's Health (NEUROFARBA), Section of Psychology, University of Florence, Florence, Italy; California University of Pennsylvania, California, PA, USA

**Keywords:** cheerfulness, humor, seriousness, bad mood, invariance, Asian, temperament

## Abstract

The State-Trait Cheerfulness Inventory (STCI) assesses latent traits and states of cheerfulness, seriousness, and bad mood to represent the temperamental basis of humor. The present study (1) tested the generalizability of the three-factor model in both state and trait versions of the STCI across European Canadian (N = 489) and first generation Chinese Canadian (N = 147) participants completing the English version of the STCI and (2) compared latent mean differences. Results indicated the confirmatory factor analyses of the three-factor model for European White participants born in Canada and Chinese participants born in China showed adequate fit for both trait and state measures. Furthermore, substantial equivalence of factor model parameters and partial scalar invariance were found for both the state and trait STCI measures. In examining latent mean differences, European White Canadian participants reported significantly higher trait cheerfulness, z = 3.30, p < .001, d = 0.84, and lower trait bad mood z = 3.25, p < .01, d = 0.80 compared to the Chinese Canadian groups. European White Canadian participants reported significantly lower state bad mood, z = 3.59, p < .001, d = 1.15, compared to the Chinese Canadian groups. Limitations and future directions based on study findings are discussed.

The conceptualization of an individual’s sense of humor as personality characteristics has been widely studied in psychological research (Martin, 2001; Ruch & Hofmann, 2012). From a trait-based psychological perspective, humor is described as the cognition, behaviors, and affect that constitute amusement, mirth, and exhilaration experienced by the individual and expressed to the surrounding environment (Ruch, 1997, 2008; Ruch, Köhler, & van Thriel, 1996). More specifically, the sense of humor can be represented as an individual’s typical behavior (i.e., trait-like characteristics) or their present state of mind (i.e., state-like characteristics) in responding to, engaging in, or producing humor (Ruch et al., 1996). The variability between and within persons for readiness to engage in humor demonstrates specific traits and states boosting or decreasing an individual’s threshold for amusement (Ruch & Hofmann, 2012). Moreover, the multidimensional nature of humor suggests the need for a model that accounts for engagement in humor and humorlessness (Ruch & Hofmann, 2012).

The STCI was designed to assess latent variables of cheerfulness and bad mood as cognitive and affective tendencies, and seriousness as an attitudinal and cognitive factor (Ruch, 1997; Ruch et al., 1996; Ruch, Köhler, & van Thriel, 1997). The state-trait model of cheerfulness postulates that high cheerfulness, low seriousness, and low bad mood would contribute to exhilaration or the tendency to laugh and respond positively to humor (Hofmann, Carretero-Dios, & Carrell, 2018; Ruch et al., 1996). Ruch and colleagues (1996) defined the construct of cheerfulness as a high prevalence of cheerful mood, the tendency to laugh easily and frequently, a cheerful interaction style, and a composed view of adverse life circumstances. These tendencies allow amusement to be facilitated, but at the same time individuals who are serious and/or in a bad mood will be less inclined to express positive affect or smile at a stimulus that can be perceived as humorous (Ruch et al., 1996). The model accounts for general tendencies (i.e., traits) and present state as well, with state cheerfulness presenting positive affectivity related to feeling merry and readiness to engage in humor-related activities at the present moment (Ruch et al., 1997). Similarly, state seriousness represents a serious frame of mind and the readiness to think and communicate seriously. State bad mood represents sad mood or ill-humored mindsets, which mitigates the preference or ability to engage in humor (Ruch et al., 1997). Evidence suggests these states show more modest test-retest reliabilities compared to their trait counterparts (Ruch et al., 1996, 1997). Indeed, state measures from the STCI amalgamated showed stronger correlations with the respective traits than single state measures, further validating the importance of measuring distinct traits and states (Carretero-Dios, Eid, & Ruch, 2011).

The evidence in the literature strongly aligns with this model. Trait cheerfulness predicts positive affect and Duchenne smiling when interacting with an amusing experimenter, bloopers, and distorted photographs of the self (Beermann & Ruch, 2011; Hofmann, 2018; Ruch, 1997; Ruch & Hofmann, 2012). Trait cheerful individuals also endorsed greater resiliency, less fear of being laughed at by others, and greater habitual tendency of laughing at oneself (Hofmann, 2018; Lau, Chiesi, & Saklofske, 2019; Ruch & Proyer, 2008). Indeed, these tendencies may allow individuals to cope better under adversity (López-Benítez, Acosta, Lupiáñez, & Carretero-Dios, 2018; Papousek & Schulter, 2010; Zweyer, Velker, & Ruch, 2004). In its relations to humor, seriousness and bad mood are associated with gelotophobia (Ruch, Beermann, & Proyer, 2009; Ruch, Proyer, Esser, & Mitrache, 2011). Depressed patients showed lower cheerfulness, higher seriousness, and greater bad mood compared to healthy control counterparts, suggesting the role of these traits in affecting the threshold of experiencing amusement (Falkenberg, Jarmuzek, Bartels, & Wild, 2011). The utility of the measure in capturing these important characteristics fundamental to humor has led to translation in over 10 languages utilized across research settings and humor-related interventions (Hofmann et al., 2018; Lau, Chiesi, Hofmann, Ruch, & Saklofske, 2019; Ruch & Hofmann, 2017; Ruch, Hofmann, Rusch, & Stolz, 2018).

Most recently, numerous studies in Chinese cultures attempted to replicate findings in Western cultures showing that possessing a benign and positive sense of humor enhances psychological well-being (for a review, see Yue, 2017). Whereas Lau, Chiesi, Saklofske, and Yan (2020) have conducted invariance studies using the STCI Chinese version, the English version of the scale could be used with Chinese participants who reside in Western cultures (e.g., Asian Americans). As such, it is important to provide evidence of the comparability of the three-factor model for European White and Asian individuals completing the measures in English. Before the STCI is shown invariant between these groups, future studies in humor and acculturation may be biased (Byrne, 2012; Byrne & Campbell, 1999). Specifically, the state-trait model of cheerfulness cannot be considered a “temperamental basis” reflecting the universality of emotions before it is shown to be invariant across cultures (Byrne & Campbell, 1999). The Chinese version of the STCI trait form has been translated and the three-factor structure has been validated with participants residing in Mainland China (Chen, Ruch, & Li, 2017). In addition, partial metric invariance for the three-factor model was found between participants residing in Canada completing the English version and participants residing in China completing the Chinese version (Lau et al., 2020).

At present, there is no evidence to support the measurement invariance for Chinese participants who reside in Western cultures (e.g., Asian Americans) using the English version of the STCI. Without at minimal partial measurement invariance, any mean differences between culturally different groups may be distinct differences arising from measurement properties, as opposed to meaningful cultural differences (Byrne, 2012). The testing of measurement invariance in the STCI would allow researchers to identify possible differences arising from measurement non-equivalence and eventually to distinguish them from actual cultural differences (Byrne & Campbell, 1999). In sum, testing measurement invariance for the state-trait model of cheerfulness will provide a foundational basis for studying the temperamental basis of humor for Chinese North Americans.

## Objectives

The aim of the present study was to test (1) the generalizability of the three-factor model in both state and trait versions of the state-trait model of cheerfulness across European Canadian and Chinese Canadian participants completing the English version of the STCI and (2) to compare latent mean differences. Measurement invariance analyses of the temperamental basis of humor can provide a solid foundation in ruling out measurement artifacts when interpreting findings for future cross-cultural studies that utilize the STCI.

## Method

### Participants and Procedure

Undergraduate students who are enrolled with English as their primary language from a large Canadian university were invited to participate in the study. Upon signing up for the study, participants were instructed to read the online consent form, complete the questionnaires, and later debriefed upon completion. Participation in the study was voluntary and participants received a credit towards their psychology course. Previous findings showed first-generation Asian Americans have views less associated with independent Western worldviews and lesser U.S. assimilation compared to second-generation Asian groups (Benet-Martínez & Karakitapoglu-Aygün, 2003). Given the nature of the research question, participants who were born in Canada and identified as European White and participants who were born in China who identified as Asian were directly compared. The European White sample born in Canada consists of 489 participants (ages ranged from 17 to 36 years; *M* = 18.96, *SD* = 2.20; 69.9% females) and the Chinese sample born in China consists of 147 participants (ages ranged from 17 to 24 years; *M* = 19.62, *SD* = 1.73; 70.7% females). The study was approved by the university’s local institutional review board.

### Measures

#### State-Trait Cheerfulness Inventory—Trait Version

The standard version of the State Trait Cheerfulness Inventory—Trait Version (Hofmann et al., 2018; Ruch et al., 1996) is comprised of 60 items providing scores on three factors relating to the theoretically-derived temperamental basis of sense of humor (i.e., cheerfulness, seriousness, bad mood). The constructs are measured on a four-point Likert-style scale ranging from 1 to 4 (1 = strongly disagree; 4 = strongly agree).

#### State-Trait Cheerfulness Inventory—State Version

The State Trait Cheerfulness Inventory—State Version (STCI-S30; Ruch et al., 1997) was designed to measure cheerfulness, seriousness, and bad mood as current states. The standard version is comprised of 30 items, with 10 items measuring each factor, and respondents utilized a four-point scale (1 = strongly disagree, 4 = strongly agree). Each factor has subcategories representative of the global latent states for cheerfulness (i.e., cheerful mood and hilarity), seriousness (i.e., earnest, pensiveness, soberness), and bad mood (i.e., sadness/melancholy, ill-humor). Ruch and colleagues (1997) published several versions of the STCI state version (e.g., “today,” “the past hour”), but the present study assessed current state (i.e., “right now”).

### Data Analysis

The 60 items of the STCI-T60 and the 30 items of the STCI-S30 were subjected to two separate single-group confirmatory factor analyses (CFA) with maximum likelihood estimation using item parcels to test the three-factor structure proposed by Ruch and colleagues (1996, 1997). The parceling procedure was applied based on the theoretical model by Ruch and colleagues (1996, 1997) to lower measurement error and manage any inherent non-normality from single item distributions (Gribbons & Hocevar, 1998; Little, Cunningham, Shahar, & Widaman, 2002). Other researchers have provided adequate justification for the use of these parcels (e.g., Carretero-Dios, Benítez, Delgado-Rico, Ruch, & López-Benítez, 2014; Hofmann et al., 2018; Ruch et al., 1996, 1997). Unidimensionality has been verified based on parallel analysis prior to item parcelling (Ferrando & Lorenzo-Seva, 2017). Detailed descriptions and examples of items for each parcel are provided in the Appendix.

Hu and Bentler (1999) suggested that model fit should be evaluated using the Root Mean Square Error of Approximation (RMSEA), Tucker-Lewis Index (TLI) and Comparative Fit Index (CFI). Byrne (2001, 2012) indicated a RMSEA value of approximately 0.10 and 0.06 would suggest moderate and excellent model fit, respectively. A CFI and TLI in the range of 0.90 and 0.95 would suggest moderate and excellent model fit, respectively.

Multigroup confirmatory factor analysis (MG-CFA) was used to examine invariance allowing sequences of progressively restrictive models (Byrne, 2012; Hong, Malik, & Lee, 2003; Meredith, 1993; Steenkamp & Baumgartner, 1998). Specifically, configural, metric, scalar, and uniqueness invariance were evaluated in a successive manner. Configural invariance involved the establishment of a baseline model that acquires the equivalent pattern of parameters (i.e., same number[s] of factor[s], same items or parcels per factor) across groups. Metric invariance (i.e., weak factorial invariance) is established when the factor pattern coefficients are constrained to equality across groups, suggesting observed differences amongst parcels reveal true differences across groups (Hong et al., 2003; Steenkamp & Baumgartner, 1998). Uniqueness invariance (i.e., strict factorial invariance) is established when error terms are constrained to equality across groups. Finally, since the STCI encompasses a multidimensional structure, factor variances and covariances invariance was also tested constraining to equality covariances of the structural part of the model.

In the sequential assessment of the models, these models are typically compared using a chi-square-based likelihood ratio test. The χ^2^ statistic is greatly sensitive to loss of fit with incremental invariance restrictions in large samples and thus, the equality constraints were tested with ΔCFI value ≤ 0.01 (Byrne, 2012; Cheung & Rensvold, 2002) supplemented by a change ≤ 0.015 in RMSEA would indicate invariance (Byrne, 2012; Cheung & Rensvold, 2002). These analyses determined whether the more restrictive model demonstrated worse fit than the previously-examined, less restrictive model (Byrne, 2012; Milfont & Fischer, 2010). When poor fit emerged, partial invariance of the most restrictive model was tested through releasing some parameters according to the model of invariance being examined (i.e., factor loadings, intercept, structural covariances, residuals for evaluating metric, scalar and factor variances and covariances, and uniqueness invariance, respectively).

Through establishing scalar (i.e., strong) invariance, or at least partial scalar invariance, meaningful comparisons across groups can be conducted. These analyses reflect meaningful cultural differences between culturally different groups rather than measurement biases (Byrne, 2012). In other words, this level of invariance allows for latent mean comparison instead of comparing the respective raw means to evaluate meaningful group differences (Milfont & Fischer, 2010; Wu, Chen, & Tsai, 2009). Additionally, uniqueness (i.e. strict) invariance between the two models is desirable even if meaningful group differences are performed at a scalar level (Gregorich, 2006). For this study, once the strong and strict invariances were tested, the latent means of the STCI factors were compared. Since the mean of a latent variable cannot be directly estimated, the European Canadian group mean was fixed to zero to estimate the difference between the means (Byrne, 2001). All statistical analyses were conducted on SPSS version 25 and SPSS AMOS 5.0 (Arbuckle, 2003).

## Results

### Descriptive Statistics and Reliability

Means, standard deviations, and bivariate correlations of the STCI-T60 and STCI-S30 for European Canadians and Chinese Canadians are presented (Table 1). To examine normality of item parcels, univariate distributions were examined. For the trait version, skewness indices ranged from −0.98 to 0.42 and –0.45 to 0.37 for European and Chinese participants, respectively. Kurtosis indices for the trait version ranged from −0.56 to 1.02 and –0.69 to 1.06 for European and Chinese participants, respectively. Similarly, skewness indices for the state version ranged from −0.16 to 0.57 and –0.30 to 0.43 for European and Chinese participants, respectively. Kurtosis indices for the state version ranged from −0.65 to 0.04 and −0.35 to 0.31 for European and Chinese participants, respectively. These values suggest no significant departures from normality (Marcoulides & Hershberger, 1997; Muthén & Kaplan, 1985).

**Table 1:** Means, Standard Deviations, and Bivariate Correlates between the State-Trait-Cheerfulness-Inventory—Trait Version (STCI-T60) and the State-Trait-Cheerfulness-Inventory—State Version (STCI-S30)

	Mean (*SD*)	
Variable	EC	CC	1	2	3	4	5	6
**1. Trait Cheerfulness**	3.16 (0.45)	2.97 (0.43)	-	0.20 [−0.04, 0.44]	−0.48** [−0.63, −0.28]	0.59** [0.43, 0.70]	0.14 [−0.05, 0.32]	−0.51** [−0.64, −0.36]
**2. Trait Seriousness**	2.64 (0.37)	2.70 (0.31)	0.00 [−0.10, 0.10]	-	0.34* [0.03, 0.42]	−0.04 [−0.22, 0.15]	0.02 [−0.00, 0.08]	0.63 [0.48, 0.74]
**3. Trait Bad Mood**	2.14 (0.54)	2.30 (0.51)	−0.71** [−0.75, −0.66]	0.03 [ −0.07, 0.12]	-	−0.55** [−0.62, −0.48]	−0.02 [0.10, 0.07]	0.61** [0.55, 0.67]
**4. State Cheerfulness**	2.68 (0.64)	2.62 (0.51)	0.59** [0.52, 0.65]	0.07 [−0.03, 0.16]	−0.55** [−0.62, −0.48]	-	0.14* [0.04, 0.23]	−0.70** [−0.75, −0.65]
**5. State Seriousness**	2.75 (0.45)	2.76 (0.38)	0.10 [0.02, 0.19]	0.39** [0.30, 0.48]	−0.02 [−0.10, 0.07]	0.14** [0.04, 0.23]	-	−0.06 [−0.15, 0.03]
**6. State Bad Mood**	1.84 (0.68)	2.09 (0.66)	−0.45** [−0.53, −0.38]	−0.09 [−0.19, 0.01]	0.61** [0.55, 0.67]	−0.70**[−0.75, −0.65]	−0.06 [−0.15, 0.03]	-

*Note.* Brackets represent Bias Corrected Accelerated (BCa) 95% Confidence interval (CI) low and upper limit (Bootstrap number = 1,000). Below Diagonal = European Canadians (EC). Above diagonal = Chinese Canadians (CC).

*p < .01 (2-tailed). **p < .001 (2-tailed).

Internal consistency of the STCI-T60 were measured using Cronbach’s α coefficients for the European White Canadian sample: cheerfulness = .92, seriousness = .78, bad mood = .92. Cronbach’s α’s for the Chinese Canadian sample were as follows: cheerfulness = .89, seriousness = .74, and bad mood = .90. Similarly, for the state version of the STCI, reliability estimates for the state version for the European White sample were as follows: cheerfulness = .91, seriousness = .73, and bad mood = .93. For the Chinese participants born in China, internal consistency values were as follows: cheerfulness = .82, seriousness = .65, and bad mood = .91.

### State-Trait Cheerfulness Inventory—Trait Version

#### One-Group CFA for Model Testing

First, a preliminary single-group CFA was conducted to examine the factorial structure of the STCI-T60 for European White and Chinese Canadian participants separately. The CFA of the three-factor model for the European White participants born in Canada showed an adequate fit, χ^2^ (74) = 366.62, *p* < .001, χ^2^/*df* = 4.95, CFI = .92, TLI = 0.90, RMSEA = .09. Factor loadings ranged from .74 to .86 for cheerfulness, .54 to .69 for seriousness, and .75 to .90 for bad mood. In terms of the structural model, seriousness was not associated with either cheerfulness or bad mood while cheerfulness and bad mood were strongly negatively associated (*r* = –.78, *p* < .001). Likewise, the CFA of the three-factor trait model for the Chinese participants born in China showed an adequate fit: χ^2^ (74) = 150.24, *p* < .001, χ^2^/*df* = 2.03, CFI = .92, TLI = 0.90, RMSEA = .08. Factor loadings ranged from .69 to .86 for cheerfulness, .42 to .70 for seriousness, and .73 to .85 for bad mood. In terms of the structural model, seriousness was positively associated with cheerfulness (*r* = .23, *p* < .05) and bad mood (*r* = .32, *p* < .05), and cheerfulness and bad mood were strongly negatively associated (*r* = –.57, *p* < .001).

#### Multi-Group CFA for Measurement Invariance Testing

Starting from the single group CFA results, the baseline model for invariance testing was defined and configural invariance was tested. The configural model with the same set of factors fixed and free parameters across groups was well-fitting (see Model 0 in Table 2) in its representation of the multigroup data (Horn, McArdle, & Mason, 1983; Meredith, 1993).

**Table 2:** Fit Statistics of the State-Trait Cheerfulness Inventory—Trait Version Invariant Models

Model	χ^2^ (*df*)	CFI	RMSEA [90% CI]	Model Comparison	∆χ^2^	∆*df*	*p*	∆CFI	∆RMSEA
Model 0: Configural invariance (unconstrained)	517.01 (148)	.919	.063 [0.057, 0.069]	-	-	-	-	-	-
Model 1: Metric invariance (measurement weights)	527.68 (159)	.919	.061 [0.055, 0.066]	Model 1 – Model 0	10.68	11	< .001	.000	.002
Model 2: Scalar invariance (measurement intercepts)	637.18 (173)	.898	.065 [0.060, 0.071]	Model 2 – Model 1	109.50	14	< .001	.022	.004
Model 2a: Partial Scalar (τCH5 free)	601.00 (172)	.906	.063 [0.057, 0.068]	Model 2a – Model 1	8.399	13	< .001	.013	.002
Model 2b: Partial Scalar (τCH5 and τSE5 free)	586.70 (171)	.909	.062 [0.057, 0.068]	Model 2b – Model 1	59.019	12	< .001	.010	.001
Model 3: Factor variances and covariances invariance (structural covariances)	618.89 (177)	.903	.063 [0.057, 0.068]	Model 3 – Model 2b	32.189	6	< .001	.006	.001
Model 4: Strict invariance (measurement error)	654.77 (191)	.898	.062 [0.057, 0.067]	Model 4 – Model 3	35.877	14	< .001	.005	−.001

*Note. df* = degrees of freedom; CFI = comparative fit index; RMSEA = root mean square error of approximation; 90% CI = 90% confidence interval around RMSEA; **∆**χ^2^ = difference in χ^2^; **∆***df* = difference in degrees of freedom; **∆**CFI = difference between CFIs; **∆**RMSEA = difference in root mean square error of approximation; τCH5 = intercept of parcel CH5; τSE5 = intercept of parcel SE5; CH = Cheerfulness; SE = Seriousness.

Metric invariance was established through constraining the equality of factor loadings. Compared with the configural model, invariance restrictions placed on factor loadings did not lead to a significant decrement in model fit (ΔCFI < 0.01; see Model 1 in Table 2). These results indicate adding invariance restrictions on factor loadings did not lead to a significant decrement in model fit compared to the configural model.

Compared to the metric invariance model, scalar invariance (see Model (2) was not established as a significant change in CFI was detected (ΔCFI = .02; see Model 2 – Model 1 in Table 2), suggesting at the same level of the latent factor, the threshold of the noninvariant parcels was different across groups. These results suggest that the endorsed levels of the underlying facets differed significantly across European White and Chinese participants. Two partial scalar models were specified with one freeing the τCH5 (i.e., CH5 representing generally cheerful interaction style) intercept of the parcel across groups (Model 2a), and the other freeing both τCH5 and τSE5 (i.e., SE5 representing preference for a sober, object-oriented communication style; Model 2b). Freeing the two specific thresholds in Model 2b led to an acceptable change in CFI for the partial scalar model when compared with the weak invariance model (ΔCFI = .01; see Model 2b – Model 1). Thus, Model 2b was selected as the final model for this step. Before establishing strict invariance, factor variances and covariances were constrained to be equal across group (Model 3) and fit indices with ∆CFI values compared to Model 2b indicated invariance. Similarly, when error terms were constrained to equality across groups (Model 4), fit indices and ΔCFI values indicated invariance.

#### Latent Mean Differences

In order to examine latent factor means between European White and Chinese Canadian students, the observed variable intercepts were constrained equal across the Canadian and Chinese groups for Model 2b (i.e., τCH5 and τSE5 free for partial scalar invariance; Byrne, 2012). In comparing latent means, constraints for CH5 and SE5 were deleted, but the cheerfulness and seriousness factors have four remaining invariant intercepts per factor (i.e., parcels). Estimates derived from this solution may be estimated accurately as the structural means model fit the data adequately with the constraints on the intercept, χ^2^ (171) = 586.70, CFI = .91, RMSEA = .06. European Canadian participants reported significantly higher cheerfulness, *z* = 3.30, *p* < .001, *d* = 0.84, and lower bad mood *z* = 3.25, *p* < .01, *d* = 0.80 compared to the Chinese Canadian groups. No significant difference between the groups was observed for seriousness, *z* = 1.12, *p* = .27.

### State-Trait Cheerfulness Inventory—State Version

#### One-Group CFA for Model Testing

Similar to the trait version, a preliminary single-group CFA was conducted to examine the factorial structure of the STCI-S30 instrument for European White and Chinese Canadians separately. The CFA of the three-factor model for European White participants born in Canada showed a good fit, χ^2^ (11) = 24.35, *p* < .05, χ^2^/*df* = 2.21, CFI = .99, TLI = 0.99, RMSEA = .05. Factor loadings ranged from .90 to .95 for state cheerfulness, .52 to .79 for state seriousness, and .88 to .96 for state bad mood. In terms of the structural model, states cheerfulness and seriousness were positively associated (*r* = .18, *p* < .01) and states cheerfulness and bad mood were strongly negatively associated (*r* = –.76, *p* < .001).

The CFA of the three-factor model for Chinese participants born in China showed a good fit, χ^2^ (12) = 25.52, *p* < .05, χ^2^/*df* = 2.126, CFI = .97, TLI = 0.94, RMSEA = .09. Factor loadings ranged from .74 to .97 for state cheerfulness, .56 to .74 for state seriousness, and .87 to .93 for state bad mood. In terms of the structural model, state seriousness was not associated with either states cheerfulness or bad mood while states cheerfulness and bad mood were strongly negatively associated (*r* = –.66, *p* < .001).

Configural invariance was tested to determine whether the number of factors for the state version were invariant across Canadian and Chinese groups. The configural model was well-fitting (see Model 0 in Table 3) in its representation of the multigroup data. Furthermore, invariance restrictions placed on measurement weights did not lead to a significant decrement in model fit compared with the configural model (ΔCFI = .009; see Model 1 – Model 0 in Table 3). These results suggest metric invariance can be established as the associations between the state factors and individual parcels were not significantly different across these two groups.

**Table 3:** Fit Statistics of the State-Trait Cheerfulness Inventory—State Version Invariant Models

Model	χ^2^ (*df*)	CFI	RMSEA [90% CI]	Model Comparison	∆χ^2^	∆*df*	*p*	∆CFI	∆RMSEA
Model 0: Configural invariance (unconstrained)	44.85 (25)	.991	.036 [0.018, 0.053]	-	-	-	-	-	-
Model 1: Metric invariance (measurement weights)	68.95 (29)	.982	.047 [0.033, 0.062]	Model 1 – Model 0	24.09	4	< .001	.009	.011
Model 2: Scalar invariance (measurement intercepts)	101.46 (36)	.970	.055 [0.042, 0.067]	Model 2 – Model 1	32.52	7	< .001	.012	.008
Model 2a: Partial Scalar (τB1 free)	96.46 (35)	.972	.054 [0.041, 0.066]	Model 2a – Model 1	27.52	6	< .001	.010	.007
Model 3: Factor variances and covariances invariance (structural covariances)	100.83 (38)	.971	.052 [0.40, 0.064]	Model 3 – Model 2a	4.37	3	< .001	.001	−.002
Model 4: Strict invariance	119.51 (45)	.965	.052 [0.041, 0.063]	Model 4 – Model 3	18.68	7	< .001	.006	.000

*Note. df* = degrees of freedom; CFI = comparative fit index; RMSEA = root mean square error of approximation; 90% CI = 90% confidence interval around RMSEA;**∆**χ^2^ = difference in χ^2^; **∆***df* = difference in degrees of freedom; **∆**CFI = Difference between CFIs; **∆**RMSEA = difference in root mean square error of approximation; B1 = intercept of parcel in the factor Bad Mood, representing a state of sad mood.

Compared to the metric invariance model, scalar invariance (Model (2) was not established as a significant change in CFI was detected (ΔCFI = .012; Model 2 – Model 1), suggesting at the same level of the latent factor, the threshold of the noninvariant parcels was different across groups. These results suggest that the endorsed levels of the underlying facets differed significantly across European White and Chinese participants. Thus, a modification of freeing one of the parcels (i.e., B1 from the factor state bad mood representing state sadness) was conducted. Freeing one specific threshold in Model 2a led to an acceptable change in CFI for the partial scalar model when compared with the metric invariance model. Thus, Model 2a was selected as the final model for this step.

Before establishing strict invariance, factor variances and covariances were constrained to be equal across group (Model 3) and the ΔCFI and ΔRMSEA values indicated invariance in comparison with Model 2a. When error terms were constrained to equality across groups (Model 4), fit indices and ΔCFI values indicated invariance when compared with Model 3.

#### Latent Mean Differences

In order to examine latent factor means between European White and Chinese Canadian students, the observed variable intercepts were constrained equal across the Canadian and Chinese groups for Model 2a (i.e., τ of parcel representing state sadness in the factor state bad mood free for partial scalar invariance; Byrne, 2012). The structural means model fit the data adequately with the constraints on the intercept, χ^2^ (35) = 96.46, CFI = .97, RMSEA = .05. Given this finding, estimates derived from this solution may be estimated accurately. European White Canadian participants reported significantly lower state bad mood compared to the Chinese Canadian groups, *z* = 3.59, *p* < .001, *d* = 1.15. No significant difference between the groups was observed for state seriousness, *z* = 0.45, *p* = .66, or state cheerfulness, *z* = –0.65, *p* = 52.

## Discussion

The present study contributed to growing literature in cross-cultural studies in humor through providing empirical evidence supporting partial strong measurement invariance of the STCI trait and state forms across first generation Chinese and European White Canadian participants. The results demonstrated that the measurement structures of cheerfulness, seriousness, and bad mood were largely equivalent across European White participants born in Canada and first generation Chinese Canadian participants born in China. Metric invariance was found for both the state and trait versions of the STCI. Upon freeing two intercepts, partial scalar invariance was found for the trait version of the STCI, which indicates cross-cultural similarity for the interpretation of the three dimensions. For the state version, upon freeing one intercept, partial scalar invariance was found. The proportion of invariant to non-invariant threshold parameters should allow fair comparisons between groups (Byrne, Shavelson, & Muthén, 1989). These findings provide evidence supporting the generalizability of the three-factor model of the temperamental basis of humor in Chinese participants (Chen et al., 2017; Lau et al., 2020).

In a previous study comparing the Chinese and English version of the STCI, noninvariant latent mean differences could not be compared given that only partial metric invariance was found (Lau et al., 2020). In the present study, the corresponding noninvariant latent factor means were examined. European White Canadian participants reported higher trait cheerfulness and lower trait bad mood compared to first generation Chinese Canadians. These results were consistent with previous findings showing Americans were more extraverted than Asians (McCrae & Terracciano, 2005). Indeed, trait cheerfulness is a narrow-level personality trait under the broader-level trait extraversion, but cheerfulness as an independent variable generally acts as a better predictor for specific humor-induced positive affect (Ruch & Hofmann, 2012). Moreover, these results align with findings that individualist cultures tend to promote positivity whereas dialectical cultures value balance of emotions (Tsai, Knutson, & Fung, 2006). As well, previous findings showed European Americans had better recall of positive affect but not negative affect, whereas Asian Americans equally recalled positive and negative affect (Wirtz, Chiu, Diener, & Oishi, 2009). Notably, Asian Canadians in this sample scored much higher in state bad mood than European White participants, but did not differ in state cheerfulness and state seriousness. Perhaps individuals of East Asian descent tend to endorse more contradictory elements in opposing emotions compared to European White North Americans who have not been exposed to dialecticism (Goetz, Spencer-Rodgers, & Peng, 2008; Spencer-Rodgers, Peng, & Wang, 2010).

The present study is not without its limitations. The sample comprised of well-educated young adults attending university and it is unclear whether these findings would generalize across different populations (i.e., difference in age, education). Furthermore, only a small sample of the Canadians recruited were second generation Chinese Canadians and of other Asian backgrounds (e.g., Korean, Japanese). These factors precluded the present study to further investigate similarities and differences between other English-speaking Asian Canadians. Moreover, the nature of humor is a multidimensional phenomenon that encompasses a function (e.g., pro-social or mean-spirited) and fulfills complex needs for the individual (e.g., engage with others, mock others). Future studies should test whether the STCI in Chinese participants predicts similar behavioral outcomes that were found in previous studies (e.g., Duchenne displays, positive benign styles of humor, frequency and intensity of laughter).

In summary, the present study extends earlier findings for a well-fitting three-factor model in the temperamental basis of humor for first generation Chinese Canadian participants. Findings from the present study provide a foundational basis for the utility of the STCI in studying the temperamental basis of humor for first generation Chinese North Americans. This study addresses concerns of extending Western models to individuals born to non-Western cultures (Gerstein & Ægisdóttir, 2012). Future studies can utilize this measure to investigate associations between the temperamental basis of humor and acculturation in North America.

## Appendix

The original Trait version 60-item set with theoretical origins from Ruch et al. (1996)

**Table:**

Facet	*N*_i_	Description	Example Item
CH1 (Cheerful mood)	5	Prevalence of cheerful mood	I am a merry person.
CH2 (Laughter threshold)	3	Low threshold for smiling and laughter	I often smile.
CH3 (Composed view)	4	Composed view of adverse life circumstances	Many adversities of everyday life actually do have a positive side.
CH4 (Broad elicitors)	4	Broad range of active elicitors of cheerfulness and smiling/laughter	I often find that the small things in everyday life are really funny and amusing.
CH5 (Cheerful Interaction)	4	Generally cheerful interaction style	I like to kid around with others.
SE1 (Serious states)	5	Prevalence of serious states	I am a serious person.
SE2 (Serious mindset)	3	Perception of even everyday happenings as important and taking it into consideration thoroughly and intensively (rather than treating them superficial)	Even seemingly trivial things have to be treated seriously and responsibly.
SE3 (Planning ahead and responsibility)	4	Tendency to plan ahead and set long-range goals (and attaining the closest possible harmony with these goals in every action and decision)	I plan my actions and make my decisions so that they are useful to me in the long run.
SE4 (Concrete activities)	4	Tendency to prefer activities for which concrete, rational reasons can be produced (thereby considering activities which don't have a specific goal as a waste of time and nonsense)	I try to spend my free time doing things as useful as possible.
SE5 (Sober style)	4	Preference for a sober, object-oriented communication style	I prefer people who communicate with deliberation and objectivity.
BM1 (Bad mood states)	5	Prevalence of bad mood states	I am often in a bad mood.
BM2 (Sadness prevalence)	6	Prevalence of sadness	Sometimes I am distressed for a very long time.
BM3 (Bad mood towards cheerfulness)	3	Sad and Ill-humored behavior in cheerfulness evoking situations, the attitudes toward such situations and the objects, persons, and roles involved	When I am distressed, even a very funny thing fails to cheer me up.
BM4 (ill-humoredness prevalence)	6	Prevalence of ill-humoredness	I am often sullen.

*Note. N*_i_ = number of items.

The original State version 30-item set with theoretical origins from Ruch et al. (1997)

**Table:**

Facet	*N*_i_	Description	Example Item
C1 (Cheerful Mood)	5	Current state of positive affectivity related to being in good spirits and feeling merry	I am cheerful.
C2 (Hilarity)	5	Readiness to engage in humor-related activities for state cheerfulness	I feel chipper.
S1 (Earnest)	3	The readiness to perceive, act, or communicate seriously	I am set for serious things.
S2 (Pensive)	4	The state of attentive, deep thought, or conducting an important task	I have important things on my mind
S3 (Sober)	3	Current serious state that applies a sober or objective perspective or style	I regard my situation objectively and soberly.
B1 (Sadness/melancholy)	5	Current sad, gloomy, and downhearted state does not enable an individual to engage in humor-related activities.	I am sad.
B2 (Ill-humored)	5	Ill-humored state (i.e., sullen, crabby) and may prefer not to engage in humor.	I am in a bad mood.

*Note. N*_i_ = number of items.
